# Neighborhood gentrification, income inequality, and cancer incidence among Hispanic/Latino adults

**DOI:** 10.1093/jncics/pkag083

**Published:** 2026-08-21

**Authors:** Yaiza Diaz-de-Durana, Corinne McDaniels-Davidson, Krista M Perreira, En Cheng, Gregory A Talavera, Linda C Gallo, Humberto Parada

**Affiliations:** School of Medicine, University of California San Diego, La Jolla, CA, United States; Division of Health Promotion and Behavioral Science, School of Public Health, San Diego State University, San Diego, CA, United States; UC San Diego Health Moores Cancer Center, La Jolla, CA, United States; Department of Social Medicine, University of North Carolina School of Medicine, Chapel Hill, NC, United States; Department of Epidemiology and Population Health, Albert Einstein College of Medicine, Bronx, NY, United States; Department of Psychology, San Diego State University, San Diego, CA, United States; Department of Psychology, San Diego State University, San Diego, CA, United States; UC San Diego Health Moores Cancer Center, La Jolla, CA, United States; Division of Epidemiology and Biostatistics, School of Public Health, San Diego State University, San Diego, CA, United States

## Abstract

**Background:**

Neighborhood gentrification leads to displacement of people, networks, and resources, and to differences in how income is distributed in the community. Few studies, however, have examined the roles of neighborhood gentrification and income inequality in relation to cancer incidence, particularly among Hispanic/Latino populations. We examined measures of neighborhood gentrification and income distribution in association with cancer risk among Hispanic/Latino adults.

**Methods:**

We included 14 303 adults (ages 18-75 years) from the Hispanic Community Health Study/Study of Latinos with geocoded addresses at baseline in 2008-2011. Incident cancers diagnosed from baseline through 2021 were ascertained via linkages with 4 state cancer registries. Neighborhood gentrification from 2000 to 2010 was assessed using the gentrification index. Income inequality in 2005-2009 was assessed using the Gini coefficient. Multivariable survey-weighted Cox regression estimated hazard ratios (HRs) and 95% confidence intervals (CIs) for the associations between quartiles of gentrification and income inequality and cancer incidence. We also conducted stratified analyses by household-level poverty (FPL), HCHS/SOL field center, and insurance status.

**Results:**

A total of 701 incident cancers were diagnosed over a mean follow-up of 10.4 (range = 0.1-13.8) years. Those in gentrification index Quartile 4 (vs Q1) had a 43% increase in cancer risk (HR = 1.43; 95% CI = 1.02 to 2.01), and those in Gini coefficient Quartile 4 (vs Q1) had a 52% increase in cancer risk (HR = 1.52; 95% CI = 1.03 to 2.26). Associations varied by health insurance status, but not by FPL or field center.

**Conclusion:**

The highest vs lowest levels of neighborhood gentrification and income inequality were associated with increases in cancer risk among Hispanic/Latino adults.

## Introduction

Cancer is a leading cause of morbidity and mortality among United States (US) Hispanic/Latino adults, with an estimated 195 000 new cases diagnosed and 50 000 cancer-related deaths annually.^1^ Although the age-adjusted incidence rate for all cancers combined is lower among Hispanic/Latino adults than among non-Hispanic White adults,^2^ Hispanic/Latino adults experience higher rates of liver,^3^ stomach,^4^ and cervical^5^ cancers. Additionally, Hispanic/Latino adults are diagnosed at more advanced stages for female breast, lung and bronchus, prostate, and stomach cancers, resulting in lower 5-year relative survival rates from these cancers.^6^ Previous studies have highlighted the complex determinants of, and racial and ethnic disparities in cancer in the US;^7^ however, few have quantified the contributions of the neighborhood to cancer risk, particularly among Hispanic/Latino adults.^8^

The neighborhood built environment and economic stability are 2 interrelated social determinants of health critical to understanding cancer health disparities.^9^ Gentrification, the process by which under-resourced, predominantly racial or ethnic minority neighborhoods experience economic investment and an influx of higher socioeconomic residents, can lead to greater income inequality and the displacement of residents, social networks, community institutions, and healthcare resources.^10^ Of direct relevance to cancer, gentrification has been shown to displace people and networks as well as resources that provide health education and timely screening, diagnosis, and treatment of cancer.^11-16^ Gentrification also leads to increased stress, which may result in physiological effects that predispose individuals to cancer.^17^ Furthermore, among those who are able to remain in gentrified neighborhoods, increased financial strain resulting from higher housing prices may increase barriers to high-quality health care, which can result in lower utilization of cancer screening and delayed cancer treatment.^13^ Thus, gentrification and income inequality may exacerbate cancer disparities, although little empirical evidence supporting this hypothesis is available.^18^^,^^19^

Studies of gentrification and cancer outcomes have primarily focused on African American or Black communities in the US. In these populations, gentrification has been shown to be associated with worse cancer outcomes among those living in highly gentrified neighborhoods.^18-20^ Studies examining neighborhood socioeconomic status (nSES) have also primarily focused on Black or African American communities and have consistently shown that lower nSES is associated with greater cancer mortality.^21-23^ Two studies conducted among Hispanic/Latino adults reported that those living in low nSES communities experienced higher rates of cancer mortality as compared to those living in high nSES communities.^24^^,^^25^ However, the social compositions of Hispanic/Latino communities differ from those of African American or Black communities, potentially altering how gentrification and inequality affect health. For example, Hispanic neighborhoods often function as ethnic enclaves that promote social cohesion and provide sources of emotional and instrumental support, potentially buffering some disadvantages; however, Hispanic/Latino populations also face structural barriers such as limited healthcare access and economic vulnerability, which may have distinct health on health impacts.^26^^,^^27^ No study, however, has examined gentrification and neighborhood income distribution in association with cancer incidence among Hispanic/Latino adults.

The objectives of this study were to examine the associations between neighborhood gentrification over a 10-year period prior to baseline and income distribution at baseline and cancer incidence over a mean follow-up of 10 years among Hispanic/Latino adults from the Hispanic Community Health Study/Study of Latinos (HCHS/SOL). We also examined whether these associations varied by household-level SES and insurance status. We hypothesized that greater levels of gentrification and income inequality would be associated with increased risk of cancer and that greater household-level SES and having health insurance would mitigate the negative effects of gentrification and income inequality on cancer risk. We also examined associations by HCHS/SOL field center given the geographically and socially heterogeneous cities included in this study.

## Methods

### Study setting and study population

This study used resources from the HCHS/SOL^28^ and the HCHS/SOL *Onco-SOL* Ancillary Study.^29^ HCHS/SOL is a community-based prospective cohort study established to evaluate the factors associated with cardiovascular disease and other chronic diseases among 16 415 Hispanic/Latino adults enrolled in 2008-2011 (Visit 1, baseline) across 4 field centers located in the Bronx, NY; Chicago, IL, Miami, FL, and San Diego, CA. As previously described,^28^^,^^30^ the HCHS/SOL used a 2-stage area probability sampling design to ensure diversity across cultural, geographic, and socioeconomic factors, as well as Hispanic/Latino backgrounds. Households were selected with stratification based on Hispanic/Latino concentration and nSES, with additional oversampling of adults aged 45 years and older at each stage. HCHS/SOL baseline assessments consisted of an in-person clinical examination and interviewer-administered questionnaires designed to assess sociodemographic characteristics and disease risk factors. The *Onco-SOL* was an HCHS/SOL ancillary study with the objectives of examining cancer etiology in this population through participant linkages with the state cancer registries in the 4 states in which the field centers were located, as described below. This study included the 14 303 HCHS/SOL participants with available geocoded baseline addresses and complete covariate data, representing 685 unique census tracts across San Diego, Miami, the Bronx, and Chicago.

All procedures performed in the HCHS/SOL involving human participants were in accordance with the ethical standards of the Institutional Review Boards of all participating institutions. All participants provided written informed consent before completing study activities.

### Cancer incidence

The primary outcomes in this study were the date of diagnosis of invasive cancer and cancer site. Incident cancers diagnosed from the HCHS/SOL baseline assessments in 2008-2011 through 2019 for CA; 2020 for FL; and 2021 for NY and IL were identified via linkages of participant records with the state cancer registries in NY, FL, IL, and CA. A total of 701 invasive cancers including 118 breast cancers, 100 prostate cancers, and 78 bronchus and lung cancers (Table S1) were diagnosed over a mean of 10.4 (min = 0.07, max = 13.80) years of follow-up among the 14 303 participants included in this study.

### Gentrification and income distribution

#### Gentrification

A gentrification index was constructed in HCHS/SOL^31^ based on the approach by Huynh and Maroko^32^ and Linton et al.^33^ as the sum of *z*-scores based on census tract-level changes between 2000 and 2010 in education (percent change in adults ages 25 years or older with a college education or greater) and nSES (change in the number of residents living below the federal poverty level, FPL, and change in the median household income). The gentrification index was calculated using the 2000 and 2010 US Census Data from the National Historical Geographic Information System and the Neighborhood Change Database, and the 5-year 2006-2010 American Community Survey (ACS). The index of gentrification ranged from −8.6 to 28.3 with higher values representing greater gentrification occurring between 2000 and 2010. The gentrification index was categorized into quartiles based on the distribution in the HCHS/SOL cohort.

#### Income distribution

The Gini coefficient,^34^ an index of income distribution, was drawn from the IPUMS National Historical Geographic Information System based on the ACS, 2005-2009.^31^ The Gini coefficient ranges from 0 to 1, with higher values indicating greater income inequality among households within a census tract.^35^ Prior to analysis, the Gini coefficient values were rescaled by multiplying each value by 10 so that possible scores ranged from 0 to 10. The Gini coefficient ranged from 0.9 to 6.7 with higher values representing greater income inequality in 2005-2009. The Gini coefficient was categorized into quartiles based on the distribution in the HCHS/SOL cohort.

### Covariates

The following covariates were used to describe the target population and were considered as potential confounders of the associations between gentrification and neighborhood income inequality and cancer risk: field center (Bronx, Chicago, Miami, San Diego), Hispanic/Latino heritage (Cuban, Dominican, Mexican, Puerto Rican, Central American, South American, and other heritage), age (continuous in years), sex (male, female), education (<high school, high school/GED, >high school), US born (born in the 50 US states or DC) or years in the US (US-yes, no and <10 years, no and ≥10 years), employment status (employed, unemployed, retired), health insurance status (yes, no), marital status (single, married or living with a partner, other), self-reported history of cancer (yes, no), and percent of the federal poverty level (FPL) calculated as the ratio of household income in US dollars given the household size by poverty level threshold (continuous). The following covariates were considered potential mediators lying on the causal pathway between gentrification/income inequality and cancer incidence: self-reported levels of total physical activity assessed using the Global Physical Activity Questionnaire (GPAQ;^36^ low, moderate, high), body mass index (BMI; <18.5, 18.5-24.9, 25.0-29.9, ≥30.0 kg/m^2^), smoking status (current, former, never smoker), and alcohol consumption (never, former, current drinker).

### Statistical analysis

All analyses used the HCHS/SOL complex survey design variables and accounted for the stratification in sample selection, cluster sampling, and weighting. We used descriptive analyses to characterize the target population overall and by quartiles of the gentrification index and quartiles of the Gini coefficient. Weighted percentages were calculated for categorical covariates and weighted means and standard errors (SEs) were calculated for continuous covariates. To estimate the hazard ratios (HRs) and 95% confidence intervals (CIs) for the associations between gentrification and neighborhood income inequality and cancer risk, we used survey Cox regression, adjusting for field center, Hispanic/Latino heritage, age, sex, education, US born and years in the US, employment status, health insurance status, and marital status. Models were not adjusted for potential mediators (ie, physical activity, BMI, smoking status, and alcohol consumption) of the associations between gentrification and income distribution and cancer risk. We examined the categorical functional forms of the gentrification and neighborhood income distribution measures to assess potential nonlinear associations and examined the continuous functional forms to assess the presumptive linear associations with tests for trend (*P*_Trend_) for cancer risk. In secondary analyses, we examined these associations overall and by FPL (>median or ≤ median), field center (Bronx, Miami, Chicago, or San Diego), and health insurance status (uninsured or insured) using stratified analyses with the continuous measures of gentrification and income distribution (to maximize power). We tested the statistical interactions (*P*_Interaction_) between the continuous measures of gentrification and income distribution and FPL, field center, and health insurance status in the covariate-adjusted Cox models. In sensitivity analyses, we examined the measures of gentrification and income distribution in a single model (ie, a co-adjusted model) given their weak correlation (Pearson *r *= 0.19).

All analyses were conducted using R version 4.2 (R Foundation for Statistical Computing)/RStudio version 2024.04.2.

## Results

The baseline characteristics of the target population are summarized in Table 1, overall and by quartiles of the gentrification index and the Gini coefficient. The mean age at baseline was 41.1 (SE = 0.3) years, over half (52.1%) were female, and over three-fourths (77.3%) were born outside of the 50 US states or DC. Among those in the highest quartile of the gentrification index, larger proportions were from the Bronx (33.1%), 61.8% were between the ages of 18 and 44 years, 51.9% were female, and 51.4% had health insurance. Among those in the highest quartile of the Gini coefficient, larger proportions were from Miami (41.5%) or the Bronx (40.6%), 58.3% were between the ages of 18 and 44 years, 50.9% were female, and 52.1% had health insurance. Lifestyle factors including physical activity, BMI, smoking, and alcohol consumption were similar across the quartiles of the gentrification index and the Gini coefficient with Quartile 4 (vs Quartile 1) having slightly higher proportions with BMI ≥30 kg/m^2^ (41.5% vs 39.2% for the gentrification index and 40.2% vs 38.8% for the Gini coefficient), and current smokers (22.9% vs 20.9% for the gentrification index and 21.7% vs 19.0% for the Gini coefficient). Compared to adults included in this study, excluded adults were more likely to be between the ages of 65 and 75 years (13.8% vs 7.9%) and female (59.2% vs 51.1%), and less likely to be born outside of the 50 US states or DC with ≥10 years of US residency (37.1% vs 51.0%) (Table S2).

**Table 1.** Target population characteristics at baseline, overall, and by quartiles of the gentrification index and the Gini coefficient of income distribution.

Characteristic		Gentrification Index^a^				Gini coefficient^b^			
	Overall	Q1	Q2	Q3	Q4	Q1	Q2	Q3	Q4
	%_weighted_	%_weighted_	%_weighted_	%_weighted_	%_weighted_	%_weighted_	%_weighted_	%_weighted_	%_weighted_
***n*_unweighted_**	14 303	3624	3558	3563	3552	3747	3570	3499	3487
**Field center**									
** Bronx**	27.5	9.2	29.8	35.9	33.1	3.1	26.3	39.0	40.6
** Chicago**	17.0	23.7	18.5	13.6	13.0	17.9	17.4	17.2	15.5
** Miami**	28.5	38.0	25.9	27.1	23.9	18.5	24.9	28.6	41.5
** San Diego**	27.1	29.2	25.8	23.4	29.9	60.5	31.4	15.2	2.5
**Age (years)**									
** 18-44**	59.8	58.5	59.3	59.3	61.8	62.3	59.7	59.0	58.3
** 45-64**	32.3	33.1	32.4	32.4	31.3	30.2	33.2	32.7	32.9
** 65-75**	7.9	8.4	8.2	8.3	6.9	7.4	7.2	8.3	8.8
**Sex**									
** Female**	51.1	51.1	51.1	50.3	51.9	49.9	52.0	51.7	50.9
** Male**	48.9	48.9	48.9	49.7	48.1	50.1	48.0	48.3	49.1
**Hispanic/Latino heritage**									
** Dominican**	9.5	3.5	10.0	12.0	11.8	1.5	10.5	12.7	12.7
** Central American**	7.4	6.1	7.6	7.9	7.8	5.9	6.5	5.7	11.2
** Cuban**	19.1	28.4	16.8	17.9	14.6	12.4	17.2	23.9	23.0
** Mexican**	38.9	45.1	39.2	33.8	38.0	69.8	40.7	28.6	17.7
** Puerto Rican**	15.9	8.8	16.3	19.8	17.9	4.9	16.8	19.8	21.5
** South American**	5.1	5.0	6.1	4.5	4.7	2.4	4.4	4.7	8.6
** Other**	4.2	3.1	4.1	4.1	5.3	3.1	3.8	4.6	5.4
**Country of birth**									
** Born in the US 50 states/DC**	22.8	19.5	23.0	22.2	25.9	25.1	23.7	21.6	20.8
** Not born in the US 50 states/DC**									
** Duration of US residency**									
** <10 years**	26.2	30.9	26.2	24.2	24.2	24.6	22.7	27.2	30.4
** ≥10 years**	51.0	49.6	50.8	53.6	49.9	50.3	53.6	51.2	48.8
**Education**									
** Less than high school**	31.0	28.7	29.7	33.5	31.7	28.8	29.2	33.6	32.3
** High school or GED**	28.1	28.8	28.6	28.1	27.0	28.0	28.2	27.9	28.2
** Greater than high school**	40.9	42.5	41.6	38.3	41.3	43.2	42.6	38.5	39.5
**Employment status**									
** Employed**	53.2	53.0	54.5	52.3	52.8	55.4	53.4	51.0	52.9
** Unemployed**	38.8	40.4	37.2	38.5	39.3	38.3	38.5	40.7	37.7
** Retired**	8.0	6.6	8.3	9.2	7.9	6.3	8.0	8.2	9.4
**Health insurance**									
** Yes**	50.8	44.8	51.8	54.6	51.4	47.3	50.2	53.2	52.1
** No**	49.2	55.2	48.2	45.4	48.6	52.7	49.8	46.8	47.9
**Household income**									
** <$10 000**	14.6	14.2	14.0	15.3	14.8	10.5	14.5	16.1	17.1
** $10 001-$20 000**	31.7	31.0	30.4	34.4	31.0	25.9	28.9	35.3	36.4
** $20 001-$40 000**	33.5	34.3	35.0	32.3	32.3	35.0	35.8	32.3	30.9
** $40 001-$75 000**	14.6	15.0	14.6	12.8	15.9	18.5	15.8	12.9	11.2
** >$75 000**	5.7	5.4	6.0	5.3	6.1	10.1	5.0	3.5	4.4
**Marital status**									
** Single**	33.4	28.6	33.6	35.8	35.1	27.9	35.2	34.8	35.4
** Married or with partner**	50.3	54.2	50.2	47.8	49.2	57.8	49.3	48.4	45.8
** Separated, divorced, widow(er)**	16.3	17.2	16.3	16.4	15.7	14.3	15.5	16.8	18.7
**Physical activity**									
** High activity**	14.2	12.8	15.1	14.6	14.1	15.6	14.9	13.2	13.0
** Moderate activity**	45.1	45.1	45.2	43.5	45.4	43.7	43.7	46.8	46.1
** Low activity**	40.8	42.1	39.7	41.9	39.5	40.7	41.5	40.0	40.8
**BMI (kg/m^2^)**									
** <18.5**	1.1	1.3	1.1	1.1	0.9	1.5	1.0	1.2	0.8
** 18.5-24.9**	21.3	21.3	21.4	21.1	21.4	21.3	21.0	19.5	23.4
** 25.0-29.9**	37.4	38.2	38.6	36.8	36.1	38.4	37.2	38.5	35.6
** ≥30.0**	40.2	39.2	38.9	41.0	41.5	38.8	40.8	40.8	40.2
**Smoking status**									
** Never smoker**	61.4	59.9	63.9	62.1	59.5	62.2	60.9	60.8	61.6
** Former smoker**	17.5	19.2	16.3	17.0	17.7	18.8	18.6	15.9	16.7
** Current smoker**	21.1	20.9	19.8	20.9	22.9	19.0	20.5	23.4	21.7
**Alcohol consumption**									
** Never drinker**	17.6	19.8	16.8	17.7	16.4	15.5	17.4	17.2	20.4
** Former drinker**	30.0	28.6	29.8	31.9	29.4	30.6	29.9	30.8	28.5
** Current drinker**	52.4	51.5	53.3	50.3	54.2	53.9	52.7	52.0	51.1
**Self-reported history of cancer**									
** No**	96.4	96.1	96.7	96.2	96.6	97.7	95.9	96.4	95.7
** Yes**	3.6	3.9	3.3	3.8	3.4	2.3	4.1	3.6	4.3

Abbreviation: BMI = body mass index.

a Gentrification index was calculated as a sum of *z*-scores for changes in the percentage of adults aged 25+ with a college education, the number of residents below the poverty line, and the median household income. Higher values represent greater gentriﬁcation.

b Income distribution is represented by the Gini coefficient of income distribution and reflects how similar household income is across households within the census tract. It can range from 0 (perfect equality) to 10 (perfect inequality).

Abbreviations: BMI = body mass index

In Table 2 we report the results examining the associations between the measures of gentrification and income distribution and cancer risk. Compared to those in Quartile 1 of the gentrification index, those in Quartile 4 had a 43% increased risk of cancer (HR = 1.43 [95% CI = 1.02 to 2.01], *P*_Trend_ = .20). Additionally, compared to those in Quartile 1 of the Gini coefficient, those in Quartile 4 had a 52% increased risk of cancer (HR = 1.52 [95% CI = 1.03 to 2.26], *P*_Trend_ = .20). In sensitivity analyses in which we examined the gentrification index and Gini coefficient in a single model, the gentrification index Quartile 4 HR was slightly attenuated and not statistically significant (HR = 1.37 [95% CI = 0.98 to 1.92], *P*_Trend_ = .23) and the Gini coefficient Quartile 4 HR was slightly stronger in magnitude and statistically significant (HR = 1.63 [95% CI = 1.10 to 2.43], *P*_Trend_ = .21) (Table S3).

**Table 2.** Survey Cox regression HRs and 95% CIs for the associations between measures of gentrification and income inequality and cancer risk.

	Gentrification index^a^				
	Quartile 1 (−8.6, −1.2)	Quartile 2 (−1.2, 0.3)	Quartile 3 (0.3, 1.4)	Quartile 4 (1.4, 28.3)	*P*_Trend_ ^b^
**Cancer events, *n***	154	180	160	207	
**Person-years**	43 122	43 711	43 016	42 441	
**IR per 1000 py**	3.57	4.11	3.72	4.88	
**HR (95% CI)^c^**	1.00 (REF)	1.18 (0.84-1.67)	1.08 (0.78–1.48)	1.43 (1.02-2.01)	.20
	**Gini coefficient^d^**				
	**Quartile 1 (0.9, 4.0)**	**Quartile 2 (4.0, 4.4)**	**Quartile 3 (4.4, 4.7)**	**Quartile 4 (4.7, 6.7)**	***P*_Trend_** ^b^
**Cancer events, *n***	144	165	194	198	
**Person-years**	42 454	42 672	43 250	43 984	
**IR per 1000 py**	3.39	3.87	4.49	4.50	
**HR (95% CI)^c^**	1.00 (REF)	1.35 (0.89-2.05)	1.32 (0.93-1.87)	1.52 (1.03-2.26)	.20

Abbreviations: CI = confidence interval; HR = hazard ratio; IR = incidence rate; py = person-years.

a Gentrification index was calculated as a sum of z-scores for changes in the percentage of adults aged 25+ with a college education, the number of residents below the poverty line, and the median household income. Higher values represent greater gentriﬁcation.

b *P*_Trend_ is the *P* value from the Cox regression model examining the continuous measure of gentrification index or income distribution.

c Model is adjusted for age (continuous in years), sex (female, male), field center (San Diego, Chicago, Bronx, Miami), Hispanic/Latino heritage (Cuban, Dominican, Mexican, Puerto Rican, Central American, South American, Other), US born and years in the US (US-born, not US-born and <10 years, ≥10 years), education (<high school/high school/college), employment status (employed, unemployed/not retired, retired), health insurance status (yes, no), federal poverty level (continuous), and marital status (single, married or living with a partner, other).

d Income distribution is represented by the Gini coefficient of income distribution and reflects how similar household income is across households within the census tract. It can range from 0 (perfect equality) to 10 (perfect inequality).

In Table 3 we report the results examining the associations between continuous measures of gentrification and income distribution and cancer risk stratified by FPL (≤ median vs > median) and in Table 4 we report the results examining the associations between continuous measures of gentrification and income distribution and cancer risk stratified by health insurance status. For FPL, the interactions were not statistically significant for either the gentrification index (*P*_Interaction_ = .91) or income distribution (*P*_Interaction_ = .83); however, a 1-unit increase in the Gini coefficient was associated with a 4% increase in cancer risk among those with ≤ median FPL (HR = 1.04, 95% CI = 1.00 to 1.09), but not among those > median FPL (HR = 0.99, 95% CI = 0.96 to 1.04). For insurance status, the interaction was not statistically significant for the gentrification index (*P*_Interaction_ = .91) but was statistically significant for the Gini coefficient (*P*_Interaction_ = .05). A 1-unit increase in the Gini coefficient was associated with a 6% increase in cancer risk among those who were uninsured (HR = 1.06, 95% CI = 1.02 to 1.11), but not among those who were insured (HR = 1.01, 95% CI = 0.98 to 1.05). There was no evidence that estimates differed by field center for either the gentrification index (*P*_Interaction_ = .94) or the Gini coefficient (*P*_Interaction_ = .51). However, none of the associations were statistically significant within strata of field center (Table S4).

**Table 3.** Survey Cox regression HRs and 95% CIs for the associations between continuous measures of gentrification and income inequality and cancer risk by federal poverty level.

	Percent of the federal poverty level^a^		
	≤Median	>Median	*P*_Interaction_ ^b^
**Gentrification index^c^**			
** Cancer events**	322	328	
** Person-years**	78 363	78 172	
** IR per 1000 py**	4.11	4.20	
** HR (95% CI)^d^**	1.02 (0.96-1.09)	1.04 (0.96-1.13)	.91
**Gini coefficient^e^**			
** Cancer events**	322	328	
** Person-years**	78 363	78 172	
** IR per 1000 py**	4.11	4.20	
** HR (95% CI)^d^**	1.04 (1.00-1.09)	0.99 (0.96-1.04)	.83

Abbreviations: CI = confidence interval; HR = hazard ratio; IR = incidence rate; py = person-years.

a The ratio of household income in US dollars by poverty threshold as a percent.

b *P* value for multiplicative interaction from Cox regression models using continuous gentrification index or income distribution and continuous percentile of poverty.

c Gentrification index was calculated as a sum of z-scores for changes in the percentage of adults aged 25+ with a college education, the number of residents below the poverty line, and the median household income. Higher values represent greater gentriﬁcation.

d Model is adjusted for age (continuous in years), sex (female, male), field center (San Diego, Chicago, Bronx, Miami), Hispanic/Latino heritage (Cuban, Dominican, Mexican, Puerto Rican, Central American, South American, Other), US born and years in the US (US-born, not US-born and <10 years, ≥10 years), education (<high school/high school/college), employment status (employed, unemployed/not retired, retired), health insurance status (yes, no), and marital status (single, married or living with a partner, other).

e Income distribution is represented by the Gini coefficient of income distribution and reflects how similar household income is across households within the census tract. It can range from 0 (perfect equality) to 10 (perfect inequality).

**Table 4.** Survey Cox regression HRs and 95% CIs for the associations between continuous measures of gentrification and income inequality and cancer risk, by insurance status.

	Insurance status		
	Uninsured	Insured	*P*_Interaction_ ^a^
**Gentrification index^b^**			.72
** Cancer events**	215	419	
** Person-years**	74 974	77 988	
** IR per 1000 py**	2.87	5.37	
** HR (95% CI)^c^**	1.04 (0.96-1.13)	1.03 (0.97-1.10)	
**Gini coefficient^d^**			.05
** Cancer events**	215	419	
** Person-years**	75 024	77 997	
** IR per 1000 py**	2.87	5.37	
** HR (95% CI)^c^**	1.06 (1.02-1.11)	1.01 (0.98-1.05)	

Abbreviations: CI = confidence interval; HR = hazard rate; IR = incidence rate; py = person-years.

a *P* value for multiplicative interaction from Cox regression models using continuous gentrification index or income distribution and continuous percentile of poverty.

b Gentrification index was calculated as a sum of z-scores for changes in the percentage of adults aged 25+ with a college education, the number of residents below the poverty line, and the median household income. Higher values represent greater gentriﬁcation.

c Model is adjusted for age (continuous in years), sex (female, male), field center (San Diego, Chicago, Bronx, Miami), Hispanic/Latino heritage (Cuban, Dominican, Mexican, Puerto Rican, Central American, South American, Other), US born and years in the US (US-born, not US-born and <10 years, ≥10 years), education (<high school/high school/college), employment status (employed, unemployed/not retired, retired), and marital status (single, married or living with a partner, other).

d Income distribution is represented by the Gini coefficient of income distribution and reflects how similar household income is across households within the census tract. It can range from 0 (perfect equality) to 10 (perfect inequality).

## Discussion

We examined the associations between gentrification and income distribution and cancer risk among Hispanic/Latino adults of diverse backgrounds. Results indicated that cancer risk increased 43% in the highest quartile of gentrification in the 10 years prior to the baseline assessments and 52% in the highest quartile of income inequality at baseline. In a model that co-adjusted for both exposures, the estimate for the gentrification index was slightly attenuated, while the estimate for the Gini coefficient was slightly stronger in magnitude compared to the results from the models where these exposures were examined separately, suggesting that both indices may capture different underlying constructs. Household-level income and HCHS/SOL field center did not significantly modify these associations, having health insurance, however, attenuated the association between income inequality and cancer risk.

Our results align with prior work suggesting that socioeconomic and neighborhood or environmental changes in neighborhoods are associated with cancer risk.^19^^,^^35^^,^^37^ Lim et al.^37^ for example, examined the impact of residential displacement in gentrifying New York City neighborhoods and found that original residents experienced interrupted healthcare access and declines in mental health. Displacement and the threat of displacement were associated with difficulty maintaining stable relationships with providers, interruptions in continuity of care, and increased psychological distress.^37^ Such disruptions are directly relevant to cancer outcomes, as they can delay screening, hinder early detection, and reduce treatment adherence. Similarly, Smith et al^19^ studied older adults living in gentrified neighborhoods and showed that rapid socioeconomic and demographic shifts were linked to increased stress, poorer self-rated physical health, and diminished social support. Altogether, these findings underscore the potential vulnerability of those who remain in gentrified neighborhoods, particularly if they have lost sources of social support or other resources. Adding to this evidence, Pichardo et al.^35^ found that men living in more gentrified neighborhoods had higher prostate cancer incidence and worse survival, especially among African American and low‐income men. Other studies, however, have reported mixed findings with respect to gentrification. While gentrification displaces residents and damages social networks, increases discrimination, and reduces affordable housing,^12^ it can also bring improvements to the neighborhoods, resulting in reduced crime and increasing health resources.^12^^,^^13^ These risks and benefits, however, are likely unevenly distributed among individuals in these neighborhoods.

Increased gentrification and income inequality are hypothesized to increase cancer risk through several interconnected mechanisms that can be explained across the 3 domains of the Neighborhood Effects Model including the physical environment, the social environment, and the service environment.^38^ Within this framework, high stress levels associated with gentrification can alter health behaviors, increase levels of anxiety and depression, and increase the risk of other physical health problems.^15^ Additionally, neighborhoods that undergo gentrification historically experience elevated exposure to environmental health risk, increased pollution exposure, and exposure to environmental hazards.^39^ Of note, however, the index of gentrification examined in this study did not include direct measures of the physical or social environments, which limits our ability to understand these associations within the context of this model. Differences in income inequality are associated with lower neighborhood social cohesion, increased stress, and limited healthcare access,^40^ which together could result in unequal access to preventive care and treatment of chronic conditions, contributing to higher cancer risk. For example, gentrification disrupts social support networks, residents’ feelings of safety and overall neighborhood stability, destabilizing the social resources that could help mitigate some of the health risks.^19^^,^^37^ Gentrification and income inequality may also reduce access to fundamental resources like affordable housing, nutritious food, and recreational spaces, thus limiting the opportunity of physical activity and healthy dietary choices, which are both key in cancer prevention.^19^^,^^37^ Several common cancers in the cohort have established population-based screening recommendations including breast and prostate, which may suggest that barriers to health care access and utilization including cancer screening may help explain these results. This hypothesis is supported by our results examining the modifying effect of health insurance status. To our knowledge, however, this is the first study to examine the associations between gentrification and income distribution and cancer risk among Hispanic/Latino adults. Therefore, studies are needed, that extend this work by examining aspects of the environment that may mediate the associations between gentrification and income inequality and cancer risk, as well as factors that may mitigate cancer risk in this population, such as racial diversity or the preservation of ethnic enclaves.

This study had several strengths, including the large and diverse sample of Hispanic/Latino adults representing the heritage groups living in the US. This diversity allows for greater generalizability to the larger US Hispanic/Latino population, which varies considerably with respect to level of acculturation, immigration status, and duration of residency in the US. Additionally, the prospective cohort study design provides a methodologically rigorous approach with clear temporality between the exposures (changes in nSES and income inequality) and the outcome (cancer incidence). However, several limitations should be noted. First, the study included a relatively young cohort at baseline, resulting in a relatively small number of cancer cases diagnosed over the 10-year follow-up, which may reduce the statistical power to detect associations, particularly for the stratified analyses. Additionally, gentrification and income distribution were only measured at or before baseline and do not account for any changes in these neighborhood characteristics after baseline, which will be important for future studies to consider given the potential displacement of residents to lower SES neighborhoods. Using census tract-level data reflects neighborhood-level rather than individual-level exposures, which may overlook personal experiences of socioeconomic change. The gentrification and income distribution measures were based on broad socioeconomic indicators, which may not capture all relevant features of neighborhood change. In addition to examining the continuous measures of the gentrification index and the Gini coefficient, we also examined quartiles of exposure. This approach resulted in a wide range in the highest quartile of the gentrification index which may combine neighborhoods experiencing moderate to high levels of socioeconomic change with those experiencing more extreme change. Therefore, estimates for the highest quartile of the gentrification index should be interpreted as reflecting the average association among neighborhoods with the highest relative gentrification scores, rather than a homogeneous category of uniformly “very gentrified” neighborhoods. Although smoking and alcohol were treated as mediators and excluded from primary models, their self-reported measurement remains relevant to the stratified analyses as misclassification of these behaviors could bias the stratified estimates and thus the interpretation of these results if differential misclassification is related to household poverty level. The exclusion of approximately 11% of HCHS/SOL participants due to missing data may have introduced bias into our results given that those who were excluded were more likely to be older and female and less likely to be born outside of the US states or DC with ≥10 years of US residence. Lastly, our results are representative of the 4 urban cities and may not generalize to other geographic areas. Despite these limitations, our findings highlight the importance of the neighborhood and neighborhood-level risk factors for cancer risk.

## Conclusion

We examined the associations between gentrification, income distribution, and cancer risk among Hispanic/Latino adults of diverse backgrounds. Our findings indicate that cancer risk were increased in neighborhoods with the highest levels of gentrification and income inequality. Future studies should continue to evaluate the mediators of these associations to elucidate the underlying mechanisms. This research will be critical for develop interventions that promote equitable neighborhood development to mitigate cancer risk among Hispanic/Latino adults and other racial or ethnic minority populations.

## Supplementary Material

pkag083_Supplementary_Data

## Data Availability

The data underlying this article are available in BIOLINCC at https://biolincc.nhlbi.nih.gov/studies/hchssol/ and can be accessed using accession number HLB01141423a or through an approved request to the HCHS/SOL.
