# Supplementary material for: Neighborhood gentrification, income inequality, and cancer incidence among Hispanic/Latino adults
Source: JNCI Cancer Spectr. 2026 Aug 21;10(5):pkag083. doi: 10.1093/jncics/pkag083 (PMC13581445; doi:10.1093/jncics/pkag083)
Supplement: Supplementary file 1 [file pkag083_supplementary_data.zip › supplemental material_R.1.docx]

**SUPPLEMENTARY MATERIAL**

**Table S1.** Top 3 cancers diagnosed in the HCHS/SOL cohort from 2008-2011 through end of follow-up^a^, overall and by quartiles of gentrification and income distribution (*n* incident cancers = 701).

| **Cancer Primary Site** | **Overall** |  | **Gentrification Index Quartiles^b^** | | | |  | **Gini Coefficient**  **Quartiles^c^** | | | |
| --- | --- | --- | --- | --- | --- | --- | --- | --- | --- | --- | --- |
|  |  |  | **Q1** | **Q2** | **Q3** | **Q4** |  | **Q1** | **Q2** | **Q3** | **Q4** |
| *n* | 701 |  | 154 | 180 | 160 | 207 |  | 144 | 165 | 194 | 198 |
| C50 BREAST | 118 |  | 24 | 34 | 21 | 39 |  | 26 | 25 | 36 | 31 |
| C61 PROSTATE GLAND | 100 |  | <15 | 29 | 25 | 32 |  | 20 | 28 | 24 | 28 |
| C34 BRONCHUS AND LUNG | 78 |  | 17 | 22 | 19 | 20 |  | <15 | 20 | 24 | 24 |
| ^a^ End of follow-up was December 31, 2019 for the California Cancer Registry; December 31, 2020 for the Florida Cancer Registry; and December 31, 2021 for the New York State Cancer Registry and Illinois State Cancer Registry. Cancer frequencies <15 are suppressed to protect participant confidentiality.  ^b^ Gentrification Index was calculated as a sum of z-scores for changes in the percentage of adults aged 25+ with a college education, the number of residents below the poverty line, and the median household income. Higher values represent greater gentriﬁcation.  ^c^ Income distribution is represented by the Gini coefficient of income distribution and reflects how similar household income is across households within the census tract. It can range from 0 (perfect equality) to 10 (perfect inequality). | | | | | | | | | | | |

**Table S2**. Baseline characteristics of excluded adults.

| **Characteristic** | **%_weighted_** |  |
| --- | --- | --- |
| *n*_unweighted_ (%) | 1,725 (100.0) |  |
| Field Center |  |  |
| Bronx | 40.4 |  |
| Chicago | 9.9 |  |
| Miami | 39.1 |  |
| San Diego | 10.6 |  |
| Age (years) |  |  |
| 18-44 | 59.5 |  |
| 45-64 | 26.7 |  |
| 65-75 | 13.8 |  |
| Sex |  |  |
| Female | 59.2 |  |
| Male | 40.8 |  |
| Hispanic/Latino Heritage |  |  |
| Dominican | 13.9 |  |
| Central American | 9.0 |  |
| Cuban | 30.1 |  |
| Mexican | 21.1 |  |
| Puerto Rican | 17.8 |  |
| South American | 4.8 |  |
| Other | 3.1 |  |
| Country of Birth |  |  |
| Born in US 50 states/DC | 22.0 |  |
| Not born in US 50 states/DC |  |  |
| Duration of US residency |  |  |
| <10 years | 40.9 |  |
| ≥10 years | 37.1 |  |
| Education |  |  |
| Less than high school | 44.4 |  |
| High school or GED | 28.0 |  |
| Greater than high school | 27.6 |  |
| Employment status |  |  |
| Employed | 27.5 |  |
| Unemployed | 61.3 |  |
| Retired | 11.1 |  |
| Health insurance |  |  |
| Yes | 48.7 |  |
| No | 51.3 |  |
| Household Income |  |  |
| <$10,000 | 16.4 |  |
| $10,001-$20,000 | 35.4 |  |
| $20,001-$40,000 | 29.5 |  |
| $40,001-$75,000 | 13.6 |  |
| >$75,000 | 5.0 |  |
| Marital Status |  |  |
| Single | 44.5 |  |
| Married or with partner | 37.9 |  |
| Separated, divorced, widow(er) | 17.6 |  |
| Physical Activity |  |  |
| High activity | 9.2 |  |
| Moderate activity | 43.0 |  |
| Low activity | 47.7 |  |
| BMI (kg/m^2^) |  |  |
| <18.5 | 1.6 |  |
| 18.5-24.9 | 27.7 |  |
| 25.0-29.9 | 35.3 |  |
| ≥30.0 | 35.5 |  |
| Smoking status |  |  |
| Never smoker | 61.4 |  |
| Former smoker | 15.1 |  |
| Current smoker | 23.5 |  |
| Alcohol consumption |  |  |
| Never drinker | 26.0 |  |
| Former drinker | 28.9 |  |
| Current drinker | 45.2 |  |
| Self-reported history of cancer |  |  |
| No | 97.0 |  |
| Yes | 3.0 |  |
|  | | |

**Table S3**. Survey Cox regression hazard ratios (HRs) and 95% confidence intervals (CIs) for the co-adjusted associations between measures of gentrification and income inequality and cancer risk.

|  | **Gentrification Index^a^** | | | | |
| --- | --- | --- | --- | --- | --- |
|  | **Quartile 1**  **(-8.6, -1.2)** | **Quartile 2**  **(-1.2, 0.3)** | **Quartile 3**  **(0.3, 1.4)** | **Quartile 4**  **(1.4, 28.3)** | ***P*_Trend_^b^** |
| Hazard ratio (95% CI)^c^ | 1.00 (REF) | 1.14 (0.81-1.58) | 1.03 (0.73-1.45) | 1.37 (0.98-1.92) | 0.23 |
|  |  |  |  |  |  |
|  | **Gini Coefficient^d^** | | | | |
|  | **Quartile 1**  **(0.9, 4.0)** | **Quartile 2**  **(4.0, 4.4)** | **Quartile 3**  **(4.4, 4.7)** | **Quartile 4**  **(4.7, 6.7)** | ***P*_Trend_^b^** |
| Hazard ratio (95% CI)^c^ | 1.00 (REF) | 1.45 (0.96-2.18) | 1.42 (0.98-2.06) | 1.63 (1.10-2.43) | 0.21 |
| CI, confidence interval  ^a^ Gentrification Index was calculated as a sum of z-scores for changes in the percentage of adults aged 25+ with a college education, the number of residents below the poverty line, and the median household income. Higher values represent greater gentriﬁcation.  ^b^ *P*_Trend_ is the *P*-value from the Cox regression model examining the continuous measure of gentrification index or income distribution.  ^c^ Model is adjusted for age (continuous in years), sex (female, male), field center (San Diego, Chicago, Bronx, Miami), Hispanic/Latino heritage (Cuban, Dominican, Mexican, Puerto Rican, Central American, South American, Other), US born and years in the US (US-born, not US-born and <10 years, ≥10 years), education (<high school/high school/college), employment status (employed, unemployed/not retired, retired), health insurance status (yes, no), federal poverty level (continuous), and marital status (single, married or living with a partner, other).  ^d^ Income distribution is represented by the Gini coefficient of income distribution and reflects how similar household income is across households within the census tract. It can range from 0 (perfect equality) to 10 (perfect inequality). | | | | | |

**Table S4.** Survey Cox regression hazard ratios (HRs) and 95% confidence intervals (CIs) for the associations between continuous measures of gentrification and income inequality and incident cancer risk, by HCHS/SOL Field Center.

|  | **Field Center** | | | | | | |  |  |
| --- | --- | --- | --- | --- | --- | --- | --- | --- | --- |
|  | **Bronx, NY** |  | **Miami, FL** |  | **Chicago, IL** |  | **San Diego, CA** |  | ***P*_Interaction_^a^** |
| **Gentrification Index**^b^ |  |  |  |  |  |  |  |  | 0.94 |
| Cancer events | 202 |  | 164 |  | 115 |  | 153 |  |  |
| Person-years | 37,847 |  | 35,680 |  | 44,375 |  | 35,059 |  |  |
| IR per 1,000 py | 5.34 |  | 4.60 |  | 2.59 |  | 4.36 |  |  |
| Hazard ratio (95% CI)^c^ | 1.05 (0.97–1.15) |  | 1.00 (0.91–1.11) |  | 1.03 (0.93–1.14) |  | 1.03 (0.87–1.21) |  |  |
|  |  |  |  |  |  |  |  |  |  |
| **Gini Coefficient^d^** |  |  |  |  |  |  |  |  | 0.51 |
| Cancer events | 202 |  | 164 |  | 115 |  | 153 |  |  |
| Person-years | 37,847 |  | 35,680 |  | 44,375 |  | 35,059 |  |  |
| IR per 1,000 py | 5.34 |  | 4.60 |  | 2.59 |  | 4.36 |  |  |
| Hazard ratio (95% CI)^c^ | 1.03 (0.97–1.10) |  | 1.00 (0.96–1.05) |  | 1.05 (0.98–1.11) |  | 1.05 (0.98–1.12) |  |  |
| CI, confidence interval; IR, incidence rate; PYs, person-years  ^a^ *P*-value for multiplicative interaction from Cox regression models using continuous gentrification index or income distribution and continuous percentile of poverty.  ^b^ Gentrification Index was calculated as a sum of z-scores for changes in the percentage of adults aged 25+ with a college education, the number of residents below the poverty line, and the median household income. Higher values represent greater gentriﬁcation.  ^c^ Model is adjusted for age (continuous in years), sex (female, male), field center (San Diego, Chicago, Bronx, Miami), Hispanic/Latino heritage (Cuban, Dominican, Mexican, Puerto Rican, Central American, South American, Other), US born and years in the US (US-born, not US-born and <10 years, ≥10 years), education (<high school/high school/college), employment status (employed, unemployed/not retired, retired), health insurance status (yes, no), and marital status (single, married or living with a partner, other).  ^d^ Income distribution is represented by the Gini coefficient of income distribution and reflects how similar household income is across households within the census tract. It can range from 0 (perfect equality) to 10 (perfect inequality). | | | | | | | | | |
